# Optimizing Emergency Medical Service Structures Using a Rule-Based Discrete Event Simulation—A Practitioner’s Point of View

**DOI:** 10.3390/ijerph18052649

**Published:** 2021-03-05

**Authors:** Christoph Strauss, Günter Bildstein, Jana Efe, Theo Flacher, Karen Hofmann, Markus Huggler, Adrian Stämpfli, Michael Schmid, Esther Schmid, Christian Gehring, David Häske, Stephan Prückner, Jan Philipp Stock, Heiko Trentzsch

**Affiliations:** 1 IMS Institut für Modellbildung und Simulation, OST—Ostschweizer Fachhochschule, Rosenbergstrasse 59, 9001 St. Gallen, Switzerland; Adrian.Staempfli@ost.ch (A.S.); Michael.Schmid@ost.ch (M.S.); 2 Rettung St. Gallen, Mooswiesstrasse 30, 9200 Gossau, Switzerland; guenter.bildstein@rettung-sg.ch; 3 Kantonsspital Baselland, 4101 Bruderholz, Switzerland; jana.efe@ksbl.ch; 4 City of Zurich, Protection & Rescue Zurich, Weststrasse 4, 8036 Zurich, Switzerland; theo.flacher@zuerich.ch; 5 Canton of Bern, Gesundheits-, Sozial- und Integrationsdirektion, Rathausgasse 1, Postfach 3000 Bern 8, Switzerland; karen.hofmann@gef.be.ch; 6 Rettungsdienst Kantonsspital Winterthur, Brauerstrasse 20, Postfach 834, 8401 Winterthur, Switzerland; markus.huggler@ksw.ch; 7 Rettungsdienst Luzerner Kantonsspital, Spitalstrasse, 6000 Luzern 16, Switzerland; esther.schmid@luks.ch; 8 Institut für Notfallmedizin und Medizinmanagement (INM), Klinikum der Universität München, LMU München, Schillerstr. 53, 80336 München, Germany; Christian.Gehring@med.uni-muenchen.de (C.G.); Stephan.Prueckner@med.uni-muenchen.de (S.P.); Heiko.Trentzsch@med.uni-muenchen.de (H.T.); 9 Center for Public Health and Health Services Reserch, University Hospital Tübingen, Osianderstrasse 5, 72076 Tübingen, Germany; david.haeske@med.uni-tuebingen.de; 10 Department of Anesthesiology, Intensive Care Medicine, Emergency and Pain Medicine, Klinikum am Steinenberg, Steinenbergstr. 31, 72764 Reutlingen, Germany; stock_j@klin-rt.de

**Keywords:** multicopter, prehospital emergency care, emergency departments, transport of patients, tracer diagnoses, response time, fleet management, emergency medical dispatch, system dynamics, group model building

## Abstract

Many studies in research deal with optimizing emergency medical services (EMS) on both the operational and the strategic level. It is the purpose of this method-oriented article to explain the major features of “rule-based discrete event simulation” (rule-based DES), which we developed independently in Germany and Switzerland. Our rule-based DES addresses questions concerning the location and relocation of ambulances, dispatching and routing policies, and EMS interplay with other players in prehospital care. We highlight three typical use cases from a practitioner’s perspective and go into different countries’ peculiarities. We show how research results are applied to EMS and healthcare organizations to simulate and optimize specific regions in Germany and Switzerland with their strong federal structures. The rule-based DES serves as basis for decision support to improve regional emergency services’ efficiency without increasing cost. Finally, all simulation-based methods suggest normative solutions and optimize EMS’ performance within given healthcare system structures. We argue that interactions between EMS, emergency departments, and public healthcare agencies are crucial to further improving effectiveness, efficiency, and quality.

## 1. Introduction

Emergency medical services (EMS) save lives [1]. The basic requirements are short response times to medical emergencies, timely implementation of life-saving emergency medical measures at the scene of the emergency and rapid transport to the nearest suitable hospital, where definitive care can timely be provided. EMS systems are subject to permanent change and the availability of EMS to meet legislative requirements may be limited by changing transport infrastructure, increased or decreased deployment due to demographic or urban planning conditions [2], staff shortages or insufficient funding. In order to guarantee comprehensive care of the population in the event of a medical emergency and to ensure the provision of EMS care according to the best possible quality standards, planning analyses and optimizations of any EMS system are indispensable. The problem of planning and optimizing locations, rosters, or relocating EMS units (see Table 1 for the term EMS unit and nomenclature in general) just-in-time has attracted the interest of operations researchers for a long time, and mathematical optimization has a long tradition in the field. There is an excellent and detailed review about EMS, including present work and an outlook for current challenges in the field by Aringhieri et al. ([3] and references there). It addresses all aspects such as (i) static and dynamic location models, (ii) relocation models, (iii) dispatching and routing policies, and (iv) a short section on the interplay with other emergency healthcare providers.

In contrast to research with a focus on mathematical simulation and optimization methods such as integer linear programming (ILP, see chapter 2 in [3]), applied research seems to focus on discrete event simulation (DES) because of its flexibility and adaptability to local peculiarities. Anything that can be implemented, as a rule, can be simulated. For this reason, we call our method a “rule-based DES”. This includes seasonal rosters or complex dispatching policies. Using scenario analysis, optimization tasks can be handled in “what-if-scenarios”. Using our rule-based DES, we analyze different scenarios and support EMS to make decisions. Usually, we combine a statistical analysis of incident data with a rule-based DES. The latter answers concrete questions of static coverage and optimal locations for EMS’ bases. In these cases, political constraints suggest an optimization based on predefined available places—a mathematical optimization is out of scope. As we will show below, rule-based DES are perfectly suited for our purposes, but optimization results can only be validated on a relative basis. Thus, unknown optimality gaps remain the main disadvantage.

The purpose of this article is to explain the rationale and fundamentals of rule-based DES as a method and illustrate its application to typical questions we have previously answered for EMS in Germany and Switzerland. We present generic results and highlight different countries’ peculiarities. As we show, EMS in Germany and Switzerland are well positioned, but putting further pressure on improving efficiency hits fundamental limitations or may even deteriorate the system of pre-hospital care.

## 2. Materials and Methods

**Figure 1:**
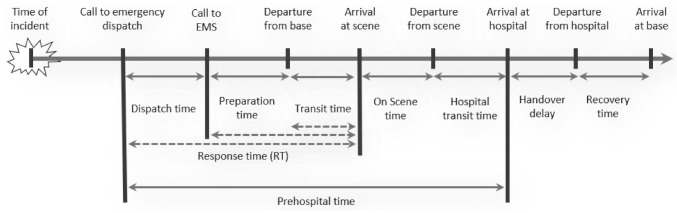
Sequence of events for each incident. The three dashed arrows represent three different definitions for response time (RT) frequently used. The case, where RT equals the transit time, is used e.g., in Bavaria (Germany); the case, where RT equals preparation time plus transit time, is standard in Switzerland, and the case, where RT starts with the call to emergency dispatch, is used in Baden-Württemberg (Germany). The definition of technical terms can be found in Table 1.

**Figure 2:**
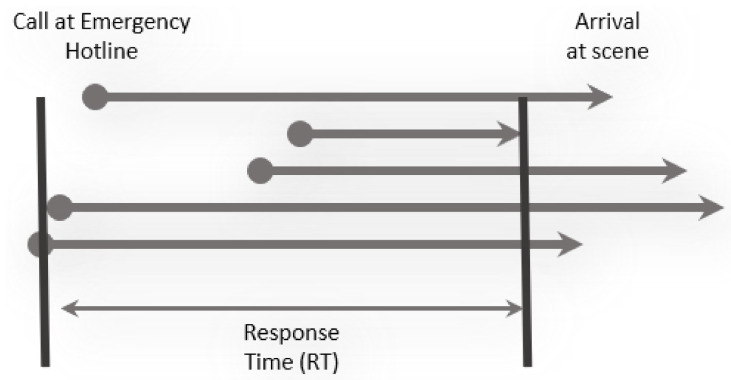
The calculation of response time (RT). In Switzerland, the maximum allowed response time (MART) is 15 min, and the maximum allowed response time compliance rate (MARTCR) is 90%. One event, e.g., a car accident, may trigger several incidents, e.g., three paramedics and two emergency doctors. The starting time of each of the four participants is indicated with a filled circle, and the duration till each arrives at the scene is indicated with an arrowhead. As communication, turnout, and traveling might take a different amount of time, the four arrows’ location and length vary. The RT is then calculated as the difference between the first call to an EMS and the first arrival at the scene. Differences across countries occur because the definitions of RT differ.

After an emergency call (see Figure 1), the dispatcher rates the priority according to Table 2 and we normally consider “P1” and “P2” as high priority. In Germany, medical associations, institutions, and organisations involved in emergency medical care recommend that at least for tracer diagnoses (TD, see Table 1), a short time to treatment in hospital improves the recovery process with high evidence [5]. Therefore, the duration to first proper treatment (see Figure 2) is measured and serves as one of the most important key performance indicators (KPI) for EMS. Because of its relevance, this duration is called the response time (RT) and Figure 1 shows its connection to various other time stamps. The maximum allowed response time (MART) and the maximum allowed response time compliance rate (MARTCR) are deduced from the RT. In Switzerland, the MART is 15 min and the MARTCR is 90%. Other time intervals mainly affect the total duration of each incident and influence the availability of EMS units: the sooner an EMS unit ends an incident, the sooner it can help with the next incident. Other parameters that affect RT and incident duration include base locations and EMS units’ rosters. Rosters indirectly influence the performance because availability is only one aspect in the case of RT. The time of call to EMS perfectly serves as event trigger and a DES is well suited to describe the dynamic behavior of the system [6,7].

### 2.1. General Foundations and Related Work

**Figure A1:**
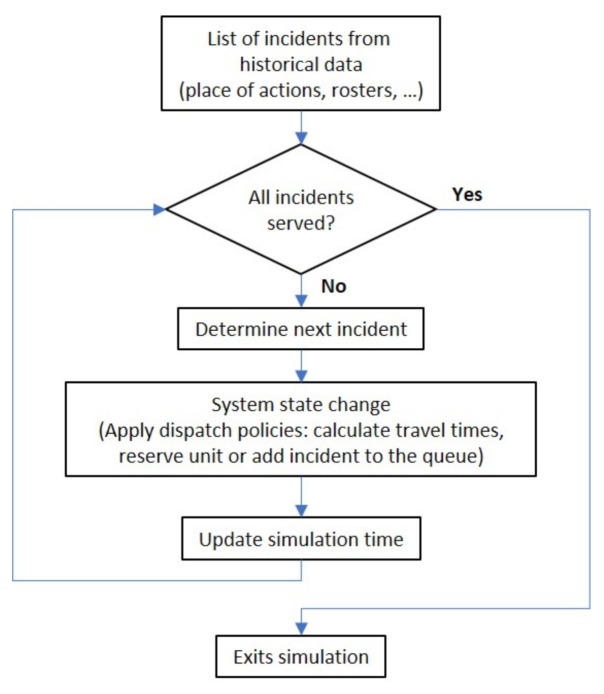
The iteration over all incidents from historic data represents the generic part of the rule-based DES. The “determination of the next incident” can be an algorithm adapted to local requirements. As an example, consider the situation where two incidents take place at the same time, require the same priority and the same type of unit. The algorithm must then select a unit according to given rules. The “system state change” is the part where the dispatch policies are applied. This part can be complex and we show the example of the multicopter project, see Figure A2 for details. The step of “updating the simulation time” just assures that the simulation moves forward.

Heyman [9] considers DES as an implementation of Markov chains, a special class of stochastic processes. From a formal point of view, this is where queues emerge as a crucial feature—in our case, patients in a queue “waiting for help” or EMS in a queue “waiting for their next incident” (see Figure A1).

**Figure 3:**
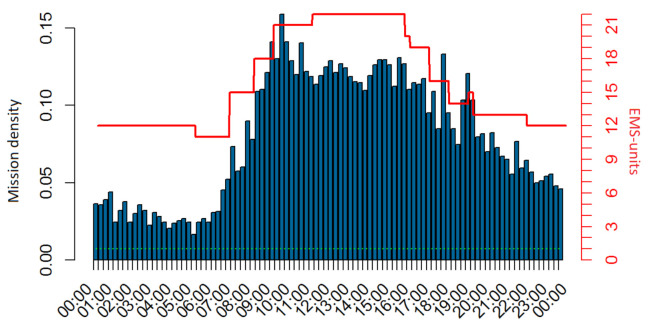
Bars show the demand per day and quarter-hour. The number of incidents is averaged over one year with about 20,000 incidents and differences between weekdays (Monday to Friday) and weekend (Saturday and Sunday) not visible. The red line was created from the rosters to show the number of available EMS units. The green line is the baseline of one EMS unit. The data originate from Switzerland, but the shape of demand is generic, see e.g., [12].

**Figure 4:**
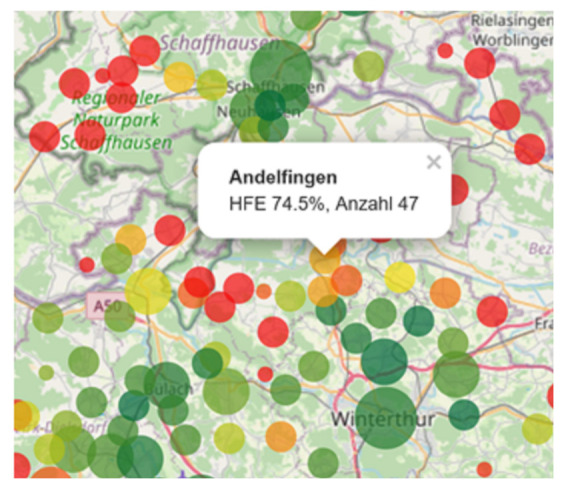
Response time compliance rate (RTCR) for a region near Zurich. As shown in the popup for Andelfingen, the RTCR is 74.5% (in German abbreviated as HFE) for 47 incidents in a 1-year period. Due to the static analysis, the responsible EMS organization requires one additional EMS unit to improve RTCR in the whole region. The color palette ranges from red (RTCR ≈60%) over orange (RTCR ≈75%) to dark green (RTCR ≈95%). The size of the circles indicates the the number of incidents (in steps 0–9, 10–99, 100–999, 1000–9999).

We could also model the different individual entities that allow us to simulate the emergence of structure (like the flock of birds’ shape [10]) or behavioral patterns in negotiations (see [11]). This results in an agent-based simulation, where the “agents” (in our case, EMS units and others) interact with each other, and the method is called agent-based modelling (ABM). We do not follow this line, because the emergency incidents provide a natural order of events with well-defined temporal (see Figure 3) and spatial (see Figure 4) random patterns. Furthermore, our analysis focusses on KPIs of the overall system, and up to now, there is no need for modelling individual strategies of paramedics, prehospital emergency physicians (PEP), or patients; we do neither intend to explain “why” emergency incidents happen nor answer questions like “what would happen if paramedics followed different strategies for interactions with their colleagues or patients.”

In contrast to ABM with a focus on single entities, mathematical optimization such as ILP calculates the global optimum at the cost of exponential growth of maximum runtime. As we need to obtain a result, ILP constitute a big risk. Furthermore, the design and implementation of ILP is much more effort and even small changes may cause a huge effort in re-implementation. Finally, mathematical optimization often contradicts local healthcare agencies’ policies of “minimal change”.

For these reasons, DES are used successfully, and we start with a short overview of related work before we explain the rule-based DES in detail. Ref. [13] report on a “generic and flexible simulation-based” tool. Their tool can deal with rescue services, interhospital patient transfers and a set of predefined hospitals which serve as destination. Furthermore, their Table 1 provides an excellent overview of related work. On the other hand, several crucial features are missing, when working with practitioners: it seems that the simulation cannot handle real data and a time resolution of 2 h is too little. Real data can be quite challenging as they are comprised of about 50,000 incidents and hundreds of EMS units. As can be seen from Figure 3, the average demand increases significantly on a 2-h basis and shifts need to be resolved at least every quarter of an hour.

A similar approach is followed by [12], who used real data over 6 months from Singapore covering more than 50,000 calls. They use first come first serve dispatch strategies, but were restricted to 2-h time intervals. On the other hand, they experimented with the dynamic reallocation of ambulances. There are two further publication from practitioners which we want to mention. Ref. [14], later continued by [15], developed a simulation tool which has now been applied to the whole of Bavaria for more than 10 years. This model is still in use and similar to what we apply in Switzerland [16]. Furthermore, ref. [17] wrote a technical paper where they presented a GIS-based interactive simulation tool which was used in Milan’s operating center. They even include further strategies for optimized dispatching. Unfortunately, no information about the present status is available.

### 2.2. Rule-Based DES in a Nutshell

Our rule-based DES merges all these features and requires at least the following input: (i) coordinates of the bases and rosters of EMS units, reflecting daily dependencies and seasonal behavior. (ii) coordinates of the place of action and (iii) priorities as shown in Figure A1. We calculate the travel time based on Open Street Map (OSM) and Open Street Map Routing Machine (OSRM). The coordinates, capacities and competences of the destination hospitals are optional input parameters. The rule-based DES then steps through all incidents (the “no”-path in Figure A1), searching for the “best” EMS unit to serve each incident.

Overall, simulation studies consist of the following three steps: (1) Based on the task, a more or less simplified model is built, and medical considerations contribute to the rules as we outline in Section 2.3. (2) We run a simulation using our historical data, as detailed in Section 2.8. The simulated history serves as crucial step in validating our rule-based DES because it seems unlikely to reproduce history based on an incorrect or oversimplified model. (3) Afterwards, any simulation of specific measures like moving a base is compared to the simulated history as explained in Section 2.9 and interactive reports are used for cummunication with customers or research partners, to check validity and results with peers (Section 2.10).

The choice of an appropriate level of simplification is one of the most challenging tasks in simulation studies. Notably, practitioners tend to “include every detail”, as they seem crucial when other stakeholders or colleagues need to be convinced of the results. Here, one of the trickiest parts concerns the EMS units to be included in the simulation and our analysis.

### 2.3. How Medical Considerations Shape the Rules of the Rule-Based DES

Up to now, the focus has been on the process (the “timing”). This section explains how medical considerations consitute the bases for other rules that we use. Any change of those rules should therefore be explainable from a medical point of view e.g., should we differentiate incidents according to priorities.

Emergency operations have different priorities and their duration depends on response time, the time it takes to assess and stabilize the patient in the field (on scene time), and the time to bring the patient to the next, appropriate hospital (hospital transit time). Thus, not only distance from the EMS base to the scene of the emergency, but also distance from the scene to the next eligible hospital, defines the time course of an EMS incident. This is the reason why not only response time but also the prehospital time, on scene time and transport time, are of critical importance in the make-up of an EMS system (see Figure 1 and Table 1).

In case of an emergency call, EMS dispatchers have to categorize its priority in order to decide what type of EMS units are required and how fast they should go to the scene (Table 2). Priority is defined by the medical condition itself. One must differentiate between the need for early life-saving intervention and fast transport to the appropriate hospital care facility. The first depends on a short response time to enable a life-saving intervention in the shortest possible time; the second aims to keep the treatment-free interval as short as possible by rapid transport to the hospital. Sudden cardiac death is the perfect example for the importance of short response time. The immediate initiation of cardio-pulmonary resuscitation (CPR) can double or triple survival from cardiac arrest. Early defibrillation has profound effect on survival and functional outcome if carried out in a timely fashion. Defibrillation within 3–5 min of collapse produce survival rates as high as (50–70)% (ERC Guideline 2020/2015, [18]).

A beneficial outcome from many medical emergencies depends on early and correct diagnosis and/or life-saving interventions that only become available at a hospital with specific expertise and infrastructure [5]. This includes, but is not limited to, computed tomography (CT), clinical chemistry, surgery, and percutaneous catheter interventions. For such cases, fast transport to the hospital is of outmost importance and pre-hospital time must be short.

Time and its effect on survival remains controversial for most conditions, but recommendations exist for time critical diagnosis providing threshold times for process measures. For example, Germany’s emergency medical associations, intuitions and organizations defined six tracer diagnoses (TD): severe traumatic brain injury, stroke, severely injured/multiple trauma, ST- elevation myocardial infarction, sudden cardiac death and sepsis [5]. These conditions have in common that there is good evidence that outcome strongly depends on timely management and guidelines with corresponding time constraints are available. So far, indicators are restricted but not limited to these six diagnoses. There are other definitions for time critical medical conditions, like the first hour quintet (FHQ), defined on the occasion of the European Resuscitation Congress in 2002 [19]. All these conditions have been attributed to a high priority. EMS legislations define time constraints for response time, but they often vary considerably in different EMS systems, even between the different states of the Federal Republic of Germany. A fair estimate is a response time of not more than 15 min in 90% of all cases. From a medical perspective, more sensible lower thresholds are desirable, but unfortunately difficult to obtain. For example, a response time of 8 min from emergency call until arrival of the first EMS unit on scene in more than 80% of the cases requires a high density of EMS units in the planning region. The associated costs are hardly manageable because response time depends on infrastructure and geographic location of emergency site and EMS bases. If the site of the emergency is in an area with excellent traffic infrastructure (i.e., motorway), ground-based EMS units travel faster and further within the given response time limit. Regions that are inaccessible to ground-based EMS units such as remote rural or alpine locations can easily be accessed, even within short periods, if air-borne rescue is available. Consequently, dispatch strategies have a major impact on RT and MARTCRs and need to be included into the simulation model.

Most hospitals are capable of handling a broad variety of medical emergencies. When it comes to fast diagnostic capacity (24/7) and special expertise/infrastructure to perform highly specialized interventions, the selection possibilities often narrow down quickly. In consequence, transport times may be extended because the way to the next neurosurgical or pediatric center is much further than to the next hospital. If patients are taken to an unsuitable hospital, this has an adverse effect on outcome and results in further time delay due to secondary transport. Therefore, the nature of the emergency determines the allocation of the patient in a given scenario, and detailed information about hospitals contributes to realistic allocation decisions.

Transport time gives an estimate of how far the scene is away from the next eligible hospital care facility. The German Social Code defines supply areas for hospitals with specialist departments for internal medicine and surgery as being accessible by private car within 30 min and for gynecology/obstetrics within 40 min. A clear advantage for the accessibility of the target hospital can be achieved with air rescue—even in the case of secondary alarming to the site of operation.

Additional delay may result from on-scene medical care or obstacles in accessing the patient. If definitive treatment in the field is not possible, the best quality indicator for the care of highly urgent medical emergencies is the pre-hospital time. For reasons of practicability, Fischer et al. agreed on the easy-to-remember but more or less arbitrarily chosen 60-min time limit as an acceptable prehospital time for TDs [5], even though certain subgroups of patients such as severely injured patients [20] and patients with acute intra-abdominal hemorrhage [21] may benefit from even shorter times to intervention. Therefore, prehospital time is also a possible KPI and is substitute for high quality in emergency with high urgency.

### 2.4. The Area to Be Considered

In contrast to large areas like Bavaria, we also face the situation, that EMS organizations with small areas and about 10,000 incidents per year ask for advice. In this case, the area needs to be restricted to the region of interest. This limited view has its own fallacies concerning analysis, simulation, and reporting, because all EMS organizations deliver services across borders. We do not consider incidents outside the region of interest, although they are part of the EMS mandate, as we want to optimize EMS for “its own area”. Considering a specific region implies to subset all events, and the response time of one specific event may not be based on the actions of a single EMS organization. Thus, there is no natural assignment of organizations to events. For a complete list of combinations of “own area”, “own responsibility”, and its fallacies, see Table A1 in Appendix B.

### 2.5. Dispatching Strategies

The most important dispatching strategy concerns priority (see Table 2), which is based on the dispatcher’s a priori rating. Depending on priority, we choose the EMS unit. We normally choose a closest-idle strategy for incidents with high priority. The easiest strategy for incidents with low priority just picks the historic vehicle if available, otherwise the incident is also served by the closest idle vehicle. These strategies may be modified, and complex ranking of queues has been implemented in a joint project which considered the Lake Constance region with its three adjacent countries, Austria, Germany, and Switzerland.

**Figure A2:**
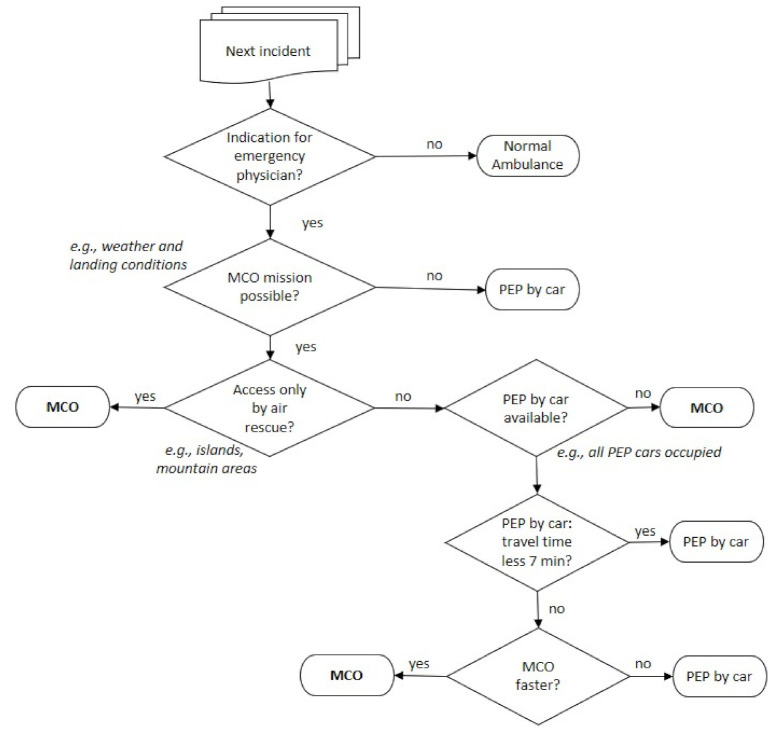
Dispatching policy for multicopters and ground based PEP units in the model regions of Germany. The algorithm was adapted for regions with air rescue helicopters. For the input (“Next incident”), see Figure A1. Parallelograms depict branching (“desicions”), oval boxes depict a “system state change”, e.g., an MCO is reserved for one incident and not available for some time (see again Figure A1).

In a project with multicopters (see example 3 in Section 3), a dispatch strategy had to be developed, which has to decide whether an emergency requires a ground-based PEP or a multicopter. We show this dispatching algorithm in Figure A2 as an implemented rule.

We also implemented a cut-off time and cut-off distance when we simulate large areas: normally, EMS units do not cross borders, and the federal structure of Germany and Switzerland even prevents EMS from crossing regional borders. In case of the project dealing with the Lake Constance region, we also applied additional rules to simulate the national as well as federal structure: If we only consider the response time compliance rate (RTCR) in the Lake Constance region, international cooperations result in +1 percentage point, while removing regional dispatching for Switzerland improves RTCR by +3 percentage points.

We frequently neglect follow-up incidents (German Folgeeinsätze) to simplify our simulations. We then assume that all EMS start at their base location, and it seems that this significantly simplified model is a good approximation for many simulations. In the case of follow-up incidents, the incident starts when the EMS unit travels back to the base location. Up to now, we do not include discontinued incidents because we do not want to optimize for false alarms, although this could be an interesting example to sensitize society for its impact on emergency medical services’ quality.

### 2.6. Simulation of Time Intervals

An important part of the simulation is the calculation of travel time to emergency sites, the transport time to hospitals, and the return trip to EMS bases. Since the EMS units are often faster than normal traffic, the parameters stored in the commercial navigation systems do not work with sufficient accuracy. Therefore, specific speed profiles in the digital road network are used for the simulations. It has to be differentiated between different types of EMS units, the priority according to Table 2 and different road types. The necessary parameters of the road networks are calculated in advance by multiple regression analyses based on real EMS data, and results are continuously validated and adjusted. The OpenStreetMap has proven to be a suitable road network and the combination with the Open Source Routing Machine ([22]) results in sufficiently accurate travel times for EMS. As our simulations call OSRM millions of times, the high-performance routing engine for the shortest routes in road networks is a key to scalable simulations.

### 2.7. The Historical Scenario

The basic inputs to the analysis and simulations are data from past incidents and the EMS units’ rosters. (The data provided by dispatch centers are subjected to ethical, legal, and privacy issues. Concepts to comply with data protection law and regulations including appropriate IT structure and safety precautions are mandatory.). Typical data sets comprise about 100,000 incidents, which ensures statistically significant results. These data describe the historical scenario on which all further changes to the EMS system are tested by simulation. These incident data have to be transferred to a standardized format with some minor but important changes: we restrict the data to a predefined area as described in Section 2.2. All incidents outside are not considered, because we do not want to optimize adjacent EMS. All incidents within the defined area which were served by external EMS are kept and we attribute all these incidents to an EMS organization defined as “external”. The RT for incidents close to the boundaries may be worse in the historical scenario, because missing data from external EMS cause longer RT, as can be seen from Figure 2. We can only resolve this issue, when a larger area is considered covering all relevant functional dependencies between different EMS organizations.

The following steps depend on the setting, but the process is always transparent to EMS or healthcare agencies: (i) Remove incidents with inconsistent or wrong time stamps. Usually, we lose less than 10 incidents in this step and we inform EMS about these missing incidents. (ii) Remove incidents with missing turnout time or response time. Usually, we lose about 5% of all incidents in this step. This may be due to aborted incidents which we do not consider. On the other hand, these incidents still create workload for EMS and we record these numbers in the beginning of each report. (iii) We remove incidents with non-complete time stamps after arrival at scene in a similar way. Usually, we lose less than about 0.1% in this step.

The availability of an EMS unit depends on the availability of the crew. The vehicle alone cannot engage in a mission unless fully crewed. Therefore, roster data must be transferred to each EMS unit. Afterwards, we calculate the events’ RT as “time, when first EMS unit arrives at scene” minus “first time of call”, as shown in Figure 2. In the end, we merge incidents, events, and EMS units into one large data structure which we call scenario. From these data, we create graphs for RTCR, as shown in Figure 4.

The analyses reveal optimization potentials and show existing weak points in an EMS system if present. Thereby, the analysis of historic data is the foundation of measures and changes, which we simulate in the following. The historical scenario relates to the historic RTCR, which allows one to answer the question of whether an EMS was able to serve its incidents, including help from outside EMS.

### 2.8. The Simulated Historical Scenario and Its Role in the Validation Process

We then simulate the historical scenario. (Our code may be available from the author upon request with the permission of the corresponding authors and institutes on the bases of mutual non-disclosure agreements.) The historical incidents and EMS units serve as unchanged input to our rule-based DES. Any differences between the historical scenario and the “simulated historical scenario” can usually be attributed to simplifications of the model and therefore require careful review to avoid factual errors within the model. This relates to the historically simulated RTCR, which indicates whether the simplifications of the model are valid. Apart from general checks of consistency (the model is in line with process and EMS’ experience), the historic RTCR serves as the most important step in internal validation, as we explain in the following. Results from the simulated historical scenario are in most cases close to the historical scenario and simulations, with improved RTCR compared to historical scenario, which are explained in many cases with the closest idle strategy, which is strictly applied in our simulations. However, these situations became rare and many simulations nowadays show a worse RTCR than stated in the historical scenario. We are able to attribute this behavior to an increased number of incidents served, increasing the utilization, and corresponding to −2 percentage points in RTCR. (In our case, there were 20,000 incidents per year and +500 incidents for one EMS organization correspond to −2 percentage points in RTCR). This behavior of “more incidents at the cost of worse RTCR” is a typical feature: when the number of EMS units increase, utilization deceases and RTCR increases. We also analyze the “prehospital time” (PT) from the call at the EMS till the arrival at the hospital, and the total service time from the call to EMS till the return to the base location (Normally, the crucial point in time is not the arrival at base location, but the time, when an EMS unit is free again,) by default, because they affect quality and the utilization, respectively.

The external validity of our DES is given, as the method has been applied in many different areas [12,13,14,15,16,17].

**Figure 5:**
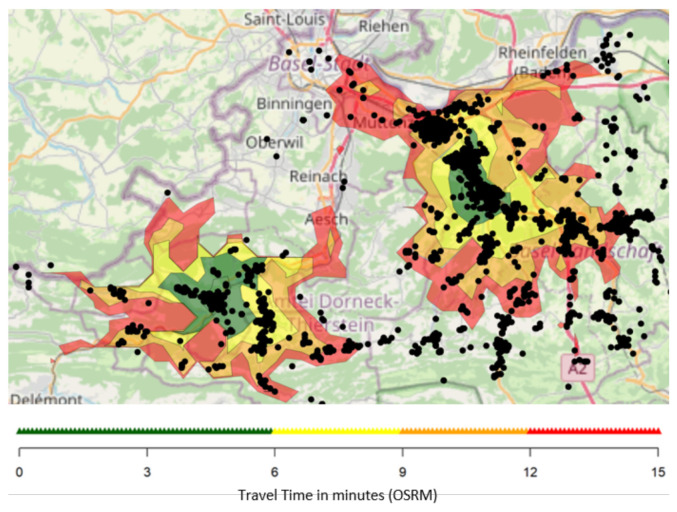
The map shows the travel time calculated with OSRM from two locations near Bale. The different colors (green, yellow, orange, and red) correspond to travel times of 0–6, 6–9, 9–12, and 12–15 min. Each black dot indicates an emergency case, and dots outside the colored regions are not reached within 15 min [22,23,24].

We always accompany the simulated historic scenario with a simulation where resources at the given bases are unlimited. If RTCR stays low in this simulation, this indicates that the base locations are not well-located or that rural areas require more EMS units to cover the area. This relates to the RTCR with unlimited resources. This “RTCR with unlimited resources” provides an upper bound to the RTCR if you are only allowed to change rosters. Results from this scenario may be compared to a static analysis, as shown in Figure 5. The advantage of a simulation “with unlimited resources” is the estimate of the RTCR, which is not accessible from the map with coverage. Furthermore, the simulations apply all relevant dispatching rules (e.g., EMS units and PEP), which ensures that time until arrival at the scene is the only free parameter entering the simulation.

### 2.9. The Simulation of Actions or the Reserve Capacity (German Rettungsmittelvorhaltung)

We then simulate any actions, such as moving a base or changing rosters, that seem promising and evaluate their impact on a quantitative basis (RTCR and the number of EMS units needed, sometimes also overtime.). This relates to the simulated RTCR, which shows whether the simulated measures show the expected effects. We analyze and validate the historical and simulated historical scenarios with the same KPI or graphs, such as shown in Figure 4, illustrating the spatial patterns of the number of incidents and the RTCR. Here, the level of “communities” is a good compromise between details and data reduction to observe important spatial patterns of deficits in coverage. An EMS organization view is mainly used for monitoring EMS. A look at single incidents is not helpful in most cases, as quality management must focus on processes. Figure 3 is also used to analyze utilization or overtime.

**Figure 6:**
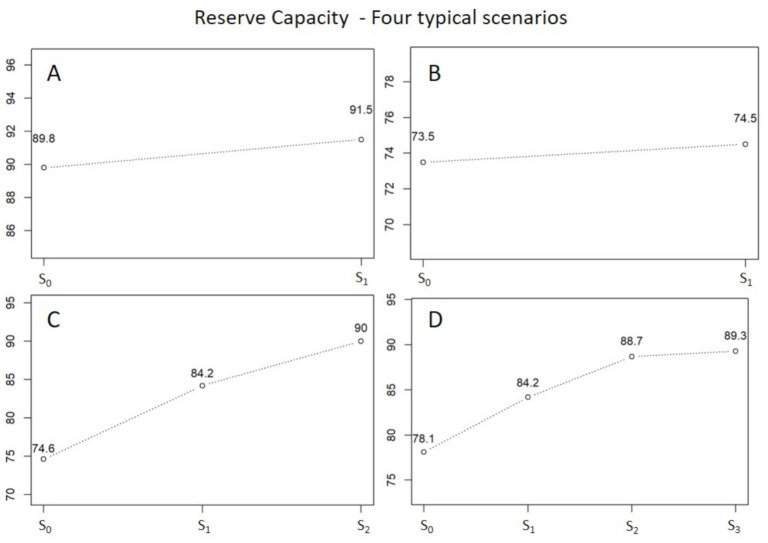
Four scenarios with simulated reserve capacity. The four different graphs A to D show four scenarios *S* in four different regions. $S_{0}$ on the x-axis indicates the simulated historical scenario; each $S_{i}$ on the x-axis indicates a scenario with “*i*” EMS units added to $S_{0}$. The y-axis shows the response time compliance rate (RTCR in %) of each scenario. The RTCR of the simulated historical scenario corresponds to the simulated historical RTCR. The four different cases show various iteration processes in the simulation of reserve capacity: a case where one EMS unit is added (**A**), a case of a rural area where no EMS unit is added because the improvement is too small (**B**), and two scenarios where 2 EMS units are added (**C**,**D**).

In some cases, we also calculate the reserve capacity (German “Rettungsmittelvorhaltung”), to simulate the number of EMS units needed to comply with a predefined MARTCR in a predefined area. Here, the base locations remain unchanged and the simulation may only change the number of EMS units. This relates to the RTCR of the simulated reserve capacity. The MARTCR is regulated by law and the resulting reserve capacity ensures help—even in rural regions, where it is difficult for EMS to operate profitably. This simulation assumes fixed bases and the simulation of the historical scenario serves as reference. If the simulated RTCR is below a certain limit defined by our partners from EMS or healthcare agencies, (1) day and night shifts are added to each base and the simulation starts. Afterwards, different simulated RTCRs are compared to add the single EMS unit with highest impact on RTCR. This step ends with one EMS unit added to the initial list of EMS units after the first iteration. Afterwards, the new RTCR is compared to the target value: if it exceeds the target value, the process stops; if it remains below the target value, the steps in (1) are repeated until (i) the target is reached or (ii) the iteration breaks, if the improvements on RTCR are too little (e.g., 1 percentage point). This is an iterative process, where we iteratively simulate with resources added on a 12-h-day- and 12-h-night-shift basis until a certain MARTCR is achieved. If the process breaks without success, adding resources is inefficient and in most cases, the simulated scenario with unlimited resources is below target as well. Alternatively, the RTCR is above MARTCR and adding resources is successful. We generate an output scenario for each step, where resources are added. The most simple analysis shows the RTCR over added resources as displayed in Figure 6 for four different areas.

Simulations always serve as one possible input to decision support and as, e.g., the simulation of reserve capacity illustrates, further input, like financial aspects, local sensitivities and others play an import role in decision making.

### 2.10. Presentation and Communication of Results

All results from simulations achieve no effect, if results are not depicted in a self-explanatory way. Therefore, we generate interactive reports as shown in Figure 4. These interactive maps with zoom- and pop-up functions can be used by EMS or healthcare agencies to define next steps. Figure 4 shows one example of such figures which include all relevant information: the color encodes the RTCR, the circles’ sizes encode the number of events in each community. The exact values are only accessible within the popup window to reduce information. EMS organizations or public healthcare agencies then use these reports to answer questions on effectivity (e.g., do the base locations still match the spatial patterns of high priority incidents?), efficiency (e.g., can we improve RTCR at unchanged cost? Does the number of EMS units match the demand?) or as a basis for decision making or discussions with other stakeholders. These questions always address quality requirements, economic aspects, or regulatory requirements.

### 2.11. General Pitfalls for Simulations

To our best knowledge, exterior constraints were responsible for unsuccessful optimization: (i) actions like moving an EMS base are not implemented because changes in infrastructure are not welcome in politics—although existing examples provide strong evidence. (ii) Private companies enter the market, further complicating the situation as incidents are split between several EMS organizations and utilization decreases. In contrast, it even happened that bases in rural areas could not be operated due to wanting paramedics. Finally, (iii) optimization of small areas misses the chance to profit from synergy effects.

## 3. Results

This section summarizes the results along three big show cases. Examples one and two are close to practice because they address EMS’ operational issues, while example 3 reports the results of a research project about how multicopters can improve the system of pre-hospital care. Here, the project needed to define potential bases, the amount of multicopters and the technical requirements for the multicopters.

All examples have the process of chapter 2 in common, namely (i) use the basic contruct of rule-based DES as depicted in Figure A1 (see Section 2.2 and Section 2.3), (ii) define the area and adapt the rules, e.g., dispatching policies to the actual needs as depicted in Figure A2 (see Section 2.4 and Section 2.5), (iii) create the historical secanrio and compare it to the simulated historical scenario (see Section 2.7 and Section 2.8) to finally reach a validated rule-based DES model. (The following examples are intended to refer to the general applicability of rule-based DES and to focus on the results we were able to achieve. For this reason, we omit the detailed steps for validation, since they are purely technical. Furthermore, they do not present anything new compared to the previous sections.)

### 3.1. Example 1: Change of Reserve Capacities (German Rettungsmittelvorhaltung)

We started with the analysis of the historic RTCR. As shown for a case near Zurich in Figure 4, the RTCR shows typical spatial patterns which reflect urban (high RTCR) and rural (low RTCR) areas. The question to be answered was, “how many EMS units divided in day- and night-shifts are needed to reach the RTCR set by public healthcare agencies?”. In the following process, we run a simulation of reserve capacity for each separated EMS organization to analyze the required number of EMS units. We show four typical cases in Figure 6, with a focus on the difficulties in the following decision process. As Figure 6 panel A shows, one has to add one EMS unit to reach the RTCR from a formal point of view—but does it make sense? Here, public healthcare agencies need to carefully balance between quality and cost and there is no measure that can tell what is right in such cases. Here, we simulate about 10,000 incidents for each EMS organization, and 0.7 percentage points relate to 70 incidents. Thus, a gain of +0.7 percentage points means that additional 70 incidents are estimated to be within 15 min when one EMS unit is added. On the other hand, nothing is said about “how much faster” these incidents are served or whether these diagnoses really need quickest possible help. Panel B shows a behavior which indicates that the simulated RTCR with unlimited resources is low, too. Adding EMS units does not make sense in this case and simulation provides a clear recommendation. Panel C is a perfect show case for simulations providing clear recommendations and public healthcare agencies suggested to add both EMS units. Finally, panel D shows a similar behavior, but RTCR saturates, indicating a problematic temporal or spatial coverage. Further improving the system requires redesigned rosters and/or relocated bases.

### 3.2. Example 2: Reallocation of EMS Bases in the Canton of St. Gallen

The EMS organizations of the canton of St. Gallen were asked to significantly improve the response time threshold from about 80% to above 90% in all regions, without increasing cost. Simulations during a feasibility study based on historic data suggested that this is possible with relocated bases and adapted rosters. As result of the simulation project, the former three independent EMS organizations merged to create a common financial basis and a re-organization project was started. After about 10 years, a MARTCR of 90% was obtained in all three regions, despite the fact that the number of incidents increased continuously (see Figure 7 from [25], updated 2020 with scaled values for the year 2020). Compared to 2013, the number of incidents increased from about 20,000 to about 27,000 per year, corresponding to a 35% growth. This only became possible, because almost all bases were relocated and new base locations as well as new rosters fitted to the demand. These changes came along with improved utilization and decreased idle time.

**Figure 7:**
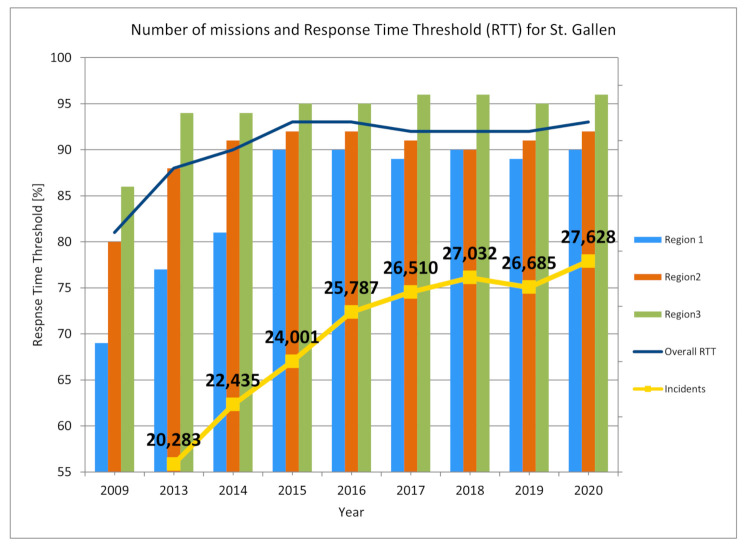
Development of number of incidents and response time threshold for the canton of St. Gallen. The graph has been first published in [25] and updated with latest estimates for the year 2020.

Figure 7 suggests that previous optimization now faces its limits for the canton of St. Gallen. We assume that the increasing number of incidents is caused by the highly dynamic environment, in which EMS operate: a growing and aging society, well-established social environments (friends, family) start to break away for older people. Furthermore, general doctors became a scarce resource, and EMS start to fill the gap. Now, the shutdown of smaller hospitals is ongoing, deteriorating the situation further.

This is somehow a typical application for our simulations, and it is frequently used by EMS organizations and public healthcare agencies. In general, these projects also discuss questions of overtime, and in one case, our simulations suggested a reduction in overtime by about −300 h per year. These predictions have been confirmed and employee satisfaction increased considerably. We also performed experiments on e.g., dynamic reallocation: if one base is empty, a neighboring base moves an EMS unit approximately in the middle of the two bases, where it awaits the next incident. Here, the key insight was that reallocation is only useful if EMS units are moved from areas with lower numbers of emergency cases to areas with a higher number of emergency cases. This contrasts with the idea of reserve capacity which intends to equally cover the whole area, and we end up with the question of proper equity principles.

### 3.3. Example 3: Multicopter as Part of Air Rescue Systems

**Figure 8:**
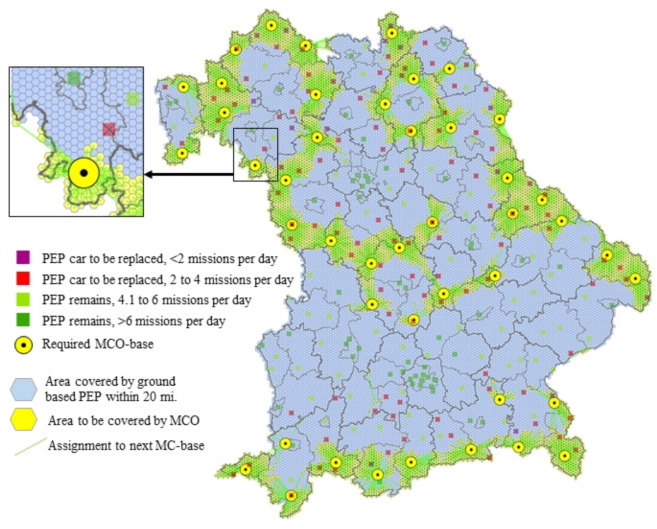
Results of the location planning for MCO using location allocation models for the region of Bavaria. MCO must achieve a complete coverage within 20 min (operation radius 24 km). These requirements were met when 114 ground-based PEP units were replaced with 43 MCO.

The multicopters (MCO) in EMS are electrically powered aircraft. Their primary task in the simulated scenario is the rapid delivery of a PEP to the emergency scene in areas that are far away from ground-based PEP sites. Due to the limited payload of MCO, patient transports are excluded as infeasible mode of operation. The simulation was used as a basic feasibility study in preparation for the first model projects with real use of multicopters in EMS [26]. On the one hand, it was about the elaboration of requirements for the currently developed aircrafts in terms of performance, range, and robustness. On the other hand, emergency operations in two regions were simulated to estimate how these new resources would affect the effectiveness and efficiency of emergency medical care. For the planning of MCO bases, extensive travel time simulations on the ground for the remaining PEP cars and airborne for rescue helicopters and MCO were initially required. Location planning was then carried out using location allocation models in order to achieve comprehensive coverage with as few multicopter locations as possible, see Figure 8 for the results. The different approaches and applications of location allocation models in the planning of EMS systems are described in detail by Khodaparasti [27].

The rule-based DES was carried out for two model regions considering the EMS incident volume of a full year. Both regions cover several districts with a total of 300,000 to 500,000 inhabitants and an annual volume of 10,000 to 15,000 EMS incidents, respectively. Not only were the incidents of the multicopters simulated, but also the incidents of all EMS units within these regions.

**Figure 9:**
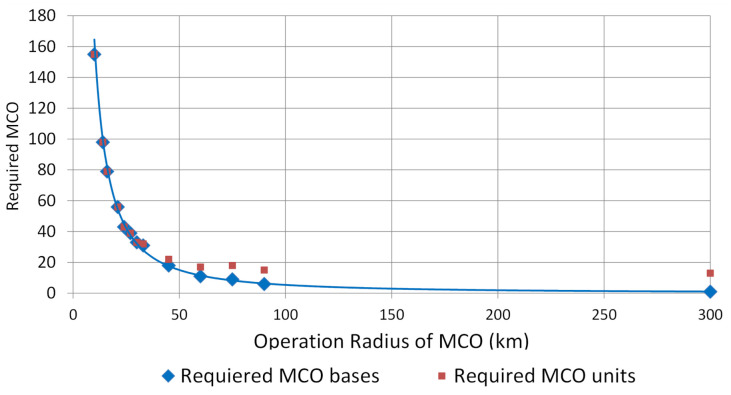
Relationship between the MCO’s radius and the required locations to achieve full coverage. The radius results from MCO’s range and speed.

Basic parameters of the simulation, such as the distance-independent parameters of the EMS incidents, were taken from previous published studies with rule-based DES and adapted to the setting of this study [28,29]. Since the range and speed of the MCOs have not yet been determined, and since these parameters were also part of the requirements analysis, the simulation of the incidents was carried out for variable speeds and ranges (see Figure 9). Furthermore, we simulated different settings, like what would happen if MCOs were implemented just in addition to the existing structures, or what would happen if several existing ground-based sites were eliminated and substituted with MCOs.

**Figure 10:**
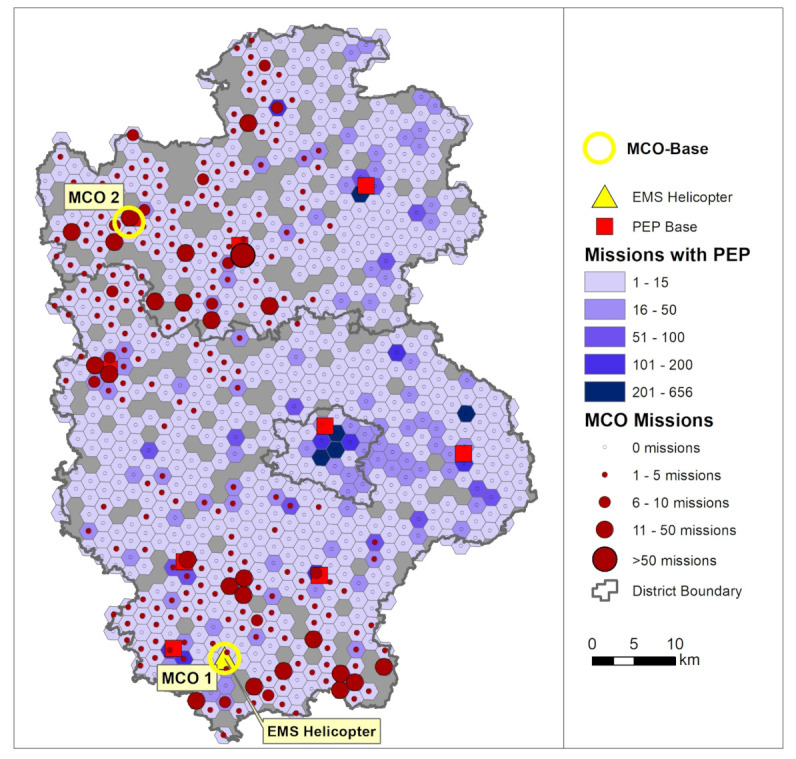
Result of the simulation model in a scenario with 2 MCO added to the areas. MCO velocity 100 km/h and MCO range 50 km. The circles show the spatial distribution of the expected MCO incidents in the Ansbach region (Bavaria). The blue coloring in the background shows the number of EMS incidents per 2km-hexagon.

The central results of the simulation were that multicopters (at speeds of about 80 km/h and a range of 50 km) as complementary rescue devices in addition to the existing structures seem capable of significantly improving ground-based PEP coverage (see Figure 10). However, the requirements are significantly higher if multicopters replace existing, poorly utilized PEP bases and must cover large supply areas. For an operation radius of 25 km, a velocity of at least 100 km/h and a minimum range of the multicopters of about 150 km are required.

## 4. Discussion

Rules-based DES are mainly used with real data and the models are always used along the steps described in Section 2, namely (i) reuse the basic model from Figure A1, (ii) adapt the rules to the current question, (iii) validate the model by comparing the simulated-historical scenario with the historical scenario. Rule-based DES’s strength is its flexibility regarding the adaptation to complex problems with different influencing variables. The whole process from importing data to the simulation of measures is transparent to all stakeholders, ensuring the acceptance of simulation results. One of the crucial steps to successful decision support through simulation is the model validation using real data. As a result, the simulated historical scenarios are part of our analysis, and changes to the historical scenarios are understood. Our work allows for deep analysis and provides a substantial gain in understanding EMS organizations’ complex operational aspects, such as incidents’ RTCR, spatial and temporal patterns, and the interplay of EMS with PEPs and hospitals. Decision support to EMS and public healthcare agencies then leads to rosters better fitted to the demand, increased utilization of EMS units (in cases with high utilization at the cost of gains in RTCR), improved RTCR (in example 2, the EMS organization started from 80% to reach 90%, while the number of incidents increased by about 35% at the same time), improved employee satisfaction, and reduced cost. We also use our simulations on a strategic level to estimate the effects of different policies, e.g., the benefit of cooperation across borders. As shown in the previous examples, the process of simulation and discussions with different stakeholders optimize efficiency and effectiveness of existing systems in the long term, as shown in [29,30]. We want to emphasize that RTCR is based solely on ED’s information (“prior RTCR”), and diagnoses/priorities might be wrong when looking back. No further analysis is possible at this time because hospital data are stored elsewhere. We hope that in the future, joint data from EMS and hospitals will be available for later analysis (“posterior RTCR”).

One of the biggest challenges which we meet regularly in our projects is the coverage of rural areas. Dynamic relocation of EMS units could be one strategy to improve coverage and equity. In contrast, this always contradicts efficiency, as EMS has the highest utilization when placed near metropolitan regions. Furthermore, these relocations are not popular with paramedics, and as forecasting is more difficult in rural areas, their economic (travel without a patient) and ecological (increase fuel consumption due to unnecessary trips) impact are negative. Increasing job variation can help with popularity, and work in that direction is in progress. Finally, we end up with a discussion about the acceptable cost of human life. Maybe insurances are one option to tackle this question in the long term, as the Swiss Department for Statistics estimates the cost for EMS to only USD 10 per person per month [31].

Another point for optimizing cost is fleet management: as specialized EMS units are expensive, it might save money to differentiate the fleet concerning its equipment. At present, there are only EMS units, patient transportation units, and PEP, but air ambulance, first responder, rapid responder, and non-professional paramedics enter the game to help in rural areas. Simulating complex fleets to estimate their impact on total cost and quality is a challenging task and dispatching according to the closest idle strategy might not always be optimal. Here, present cost structures are questioned and smaller EMS organizations might need to close because cost-effectiveness decreases further. On the strategic level, separated finances constitute a major obstacle to launch different fleets (e.g., air ambulance), or to improve across organizations or borders. Here, simulation could serve as a basis for negotiating the “rules of the game” to combine EMS and air ambulance for optimal health and financial outcome. This could also help in the case of rural areas along the way. There are also plans to separate rescue services (public interest) from interhospital patient transfers (business case), but the optimization potential and the impact on EMS’ overall cost structures, as well as fleet management, remain unclear.

One of the strategic discussions in Germany and Switzerland also address the number and locations of hospitals, and most strategies imply a reduction of hospitals while focusing on specific treatments. These changes in prehospital care directly influence the performance of EMS because the travel time increases significantly. EMS provide live-saving intervention on scene, but only qualified hospitals provide definitive care, and we suggest a common classification of hospitals according to their ability to treat various diagnoses, such as those listed as TDs.

Apart from the cost of human life, the question arises, to what extent is the present focus on RT justified? As soon as hospitals enter the game, one should also consider PT as an influencing parameter, with a significant impact on the medical outcome of pre-hospital care. Quick help with consecutive transport to an unqualified hospital (e.g., in case of a stroke) may have significant negative impact to the overall healthcare cost and steering prehospital care needs a broader focus than EMS only. In Switzerland, costs for EMS cumulated to about one billion CHF per year, whereas total health cost amount to about 60 billion CHF per year [31].

In total, there are many conflicting areas in EMS’ environment: metropolitan regions (EMS with high utilization are cost effective) vs. rural areas (EMS with low utilization are costly). Decisions about the transport destination may follow other rules in reality than in the simulation. The 24 h 7 days operation of a highly specialized care facilities can be just as unrealistic as the claim of a primary care hospital to be able to treat all cases successfully. In all these cases, simulation might be misinterpreted as decision support goes wrong or predictions are not met. However, this is not a problem of simulations in general or rule-based DES in particular, but more a situation of inadequate system boundaries. Unfortunately, these situations are hard to detect, as participants have an interest in not discussing the underlying problems in public. The negotiation on conflicting interests is closely related to a common language, which serves as a so-called boundary object [32,33]. Both authors argue that the development of a common language comes in hand with increasing systemic understanding and rule-based DES may serve as a second boundary object to negotiate on different interests in the future. As prehospital emergency care is a complex and dynamic system including EMS, emergency departments, and public healthcare agencies, a pure rule-based DES approach cannot succeed, and we suggest to use methods from system dynamics [32,34,35,36] to tackle the problem on a scientific basis. Here, we claim untapped optimization potentials, and steering pre-hospital care not only depends on medical considerations but requires regional “Emergency Visions”. These visions can be developed in a participative manner, such as group model building [36,37]. As part of this process, we seek a broad medical consensus on TDs, which serve as input to our rule-based DES. These preconditions allow estimating the impact of different models for prioritization and dispatching strategies based on common TDs, as presented in [5].

## 5. Conclusions

Rule-based DES are a good compromise between the mathematics of stochastic processes (pure DES) and ABM, because their predictions are in good agreement with real data. The model can be validated using the “simulated historical scenario”. In addition, changes can be easily implemented and anything that can be specified as a rule can be simulated. Due to rule-based DES’ reliability, results serve as decision support to improve the outcome of change-processes. Using rule-base DES this way, EMS organizations have been optimized during the last 10 years and limits emerge because of EMS’ high utilization. Further pushing technical changes such as traffic light systems, or changing organizations (like moving bases) might further improve EMS. However, we think that a shift on prehospital care (e.g., through parameters like PT) could have a much bigger impact.

Mathematical optimization frameworks such as ILP might become a tool to detect further optimization potential but serve at least as upper limit to RTCR in the future or allow to optimize on multiple objectives such as RTCR and PT. Here, the question is, “how to get the appropriate EMS unit in proper time to patients and how can we transfer these patients to the right hospital?” at minimum cost and maximized quality. Simulations are the only possibility to test costly changes without risk. The rapid development of computing power and the availability of high-quality data suggest a just-in-time calculation of undersupplied regions based on real-time travel times, including knowledge about traffic, available hospitals, and suggestions for relocations. Simulations can help to quantify the impact of these effects in advance to avoid negative unanticipated consequences. In contrast, dynamic relocation of EMS units might also result in higher inequality concerning coverage of rural areas.

As we have shown, rule-based DES is well suited to simulate and optimize operational aspects of EMS, but it reaches its limits when normative specifications are no longer realistic. Involving all stakeholders is the only way to improve on prehospital care in the future, when systemic constraints prevent further optimization. A combination of methods from operations research and system dynamics provides a guideline to push research on prehospital care.

## Figures and Tables

**Table 1:** Terms in use in this article. All authors work in German-speaking areas, and the German translation is sometimes added, because we sometimes face misleading nomenclature.

Term	Descrition
Call	One appeal or demand for EMS to drive to the place of action. In the literature often denoted “incident” or “mission”. (German Einsatz)
CP	Community paramedic (German Gemeindenotfallsanitäter)
ED	Emergency (Medical) Dispatch. The term emergency dispatch refers to a system that enhances services provided by Public Safety Answering Point (emergency) call takers, such as municipal emergency services dispatchers. Also called Emergency Medical Dispatch
EMS	Emergency Medical Services. Also known as ambulance services or paramedic services, are emergency services that provide urgent prehospital treatment and stabilisation for serious illness or injuries and transport to definitive care. If we mean the organization, we explicitly write “EMS-organization”, the EMS unit consists of the vehicle and a team that are available for a certain period of time.
FHQ	First Hour Quintet. Introduced in 2002 by the European Resuscitation Council. The abbreviation is mainly used in German and covers five key diagnoses (Stroke, Chestpain, Herz-Kreislaufstillstand, schweres Trauma, akute Atemnot), where fast aid by EMS is crucial to the outcome.
FR	First Responder. A non-professional with specialized training who is among the first to arrive and provide assistance at the scene of an emergency.
GH	Golden hour. The golden hour is a phase in prehospital care with high impact on the medical outcome [4]. From our understanding the golden hour is best represented by the prehospital time.
Incident	The total involvement for an emergency. This includes all those involved from the dispatcher to EMS to Police and fire department in the prehospital case. One incident may generate several calls. (German Ereignis)
KPI	Key Performance Indicator
KTW	Patient transport ambulance (German Krankenwagen)
MAPT	Maximum allowed prehospital time. The time desired from dispatch to arrival at the hospital. (German Prähospitalfrist)
MARTCR	Maximum allowed response time compliance rate. The number of calls that reached the maximum allowed time (German Einhaltungsgrad der Hilfsfrist)
MART	Maximum allowed response time. The time of the call to the time arrived at scene. (German Hilfsfrist)
MCO	Multicopter, a new type of air ambulance
PEP	Prehospital Emergency Physician (German Notarzt)
PT	Prehospital time. The time of the call to the time arrived at the hospital. (German Prähospitalzeit)
RR	Rapid Responder. In Switzerland these are professionals who can be called in their free time, if an emergency happens nearby.
RT	Response time. The time that a EMS unit is dispatched. This can also be the length of time from dispatch to the arrival on the scene. (German Therapiefreies Intervall)
RTCR	Response time compliance rate. The number of calls that meet the desired time allotment. (German Einhaltungsgrad für das Therapiefreie Intervall)
RTW	Ambulance vehicles (German Rettungswagen)
TD	There are six tracer diagnoses: severe traumatic brain injury, stroke, severely injured/multiple trauma, ST- elevation myocardial infarction, sudden cardiac death and sepsis [5]. These conditions have in common that there is good evidence that outcome is strongly dependent on timely management and guidelines with corresponding time constraints are available. Therefore, these diagnoses are suitable for tracing quality of care.

**Table 2:** Different priority categories in Switzerland [8]. “P” is an abbreviation for primary incident (or emergency case), “S” is an abbreviation for secondary incident (or transportation). The nomenclature may vary within Switzerland. Furthermore, names or descriptions vary slightly from Switzerland to Germany, but the principle is the same.

Priority	Description
P1	Emergency incident with traffic privileges and suspected impairment of vital functions
P2	Emergency incident without suspected impairment of vital functions
P3	Preordered incident
S1	Relocation with suspected impairment of vital functions (with or without traffic privileges (Lights, Signal))
S2	Time critical relocation without suspected impairment of vital functions
S3	Preordered relocation

## Data Availability

The data provided by dispatch centers is subjected to ethical, legal, and privacy issues. Concepts to comply with data protection law and regulations including appropriate IT-structure and safety precautions are mandatory.

## Appendix A. Flow Chart for a Rule Based DES

We show a simplified schematic for our rule-based DES. The generic part (Figure A1) basically shows a simplified time line, where each incident is finally served. In case of wanting units, an incident may be added to a queue temporarily. The application of dispatch policies is described in detail in Figure A2.

## Appendix B. Restricting the Area and its Fallacies

If we consider the different combinations of (“own area”, “own responsibility”), the following important cases arise in a natural way.

**Table A1:** “Own area” and “own responsibility” both have values “Both”, “True” or “False”.

Case	(Own Area, Own Responsibility)	Use Case
1	(B, T)	RTCR for all events where the particular EMS organization is involved.
2	(T, B)	RTCR for all events in the area of a particular EMS organization (no matter which EMS served an event).
3	(T, T)	RTCR for all events in the area of a particular EMS organization, which were served by this particular EMS organization.
4	(T, F)	RTCR for all events in the area of a particular EMS organization that were served by external EMS organizations.
5	(F, T)	RTCR for all events in the area of external EMS organizations that were served by this particular EMS organization.

There are four fallacies: (i) if we filter by EMS organization, we only keep incidents at locations where the EMS organization is responsible. Events are then selected via incidents, that is, the historic RTCR remains unchanged. In this case, the number of filtered incidents and events per organization varies depending on the number simultaneous incidents (German Simultaneinsätze). As we keep the incidents and events for all involved organizations, the sum of incidents/events over different organizations may be larger than the total number of incidents/events. If an EMS organization is simulated without help from other external EMS organizations, the historic simulated RTCR answers the question if the EMS organization is able to serve all incidents with its own resources only. (ii) If we keep all EMS organizations but only consider their own area, we only keep incidents in their own area of responsibility. This restricts incidents to a pre-defined area. (iii) Events are selected via incidents—except for the case where we do not keep all EMS units (e.g., when we only consider the incidents requiring PEPs). Here, the RT of these particular EMS units is of interest, and the RT is re-calculated on the subset of events. (iv) The selected region must contain all relevant hospitals if changes in the hospital transit time are expected. This happens if e.g., hospitals close down or hospitals nearby the place of action cannot deliver the requested treatment.
